# Stereo-Complex and Click-Chemical Bicrosslinked Amphiphilic Network Gels with Temperature/pH Response

**DOI:** 10.3390/gels9080647

**Published:** 2023-08-11

**Authors:** Wanying Yang, Jiaqi Wang, Lingjiang Jia, Jingyi Li, Shouxin Liu

**Affiliations:** Key Laboratory of Applied Surface and Colloid Chemistry, Ministry of Education, School of Chemistry and Chemical Engineering, Shaanxi Normal University, Xi’an 710119, China; yangwy15935903470@163.com (W.Y.); wjqsxdtdx@163.com (J.W.); lingjiang0711@snnu.edu.cn (L.J.); 41908194@snnu.edu.cn (J.L.)

**Keywords:** hydrogel, physicochemical double crosslinking, stereoscopic complexation, click chemistry, temperature response, pH response

## Abstract

Stimulus-responsive hydrogels have been widely used in the field of drug delivery because of their three-dimensional pore size and the ability to change the drug release rate with the change in external environment. In this paper, the temperature-sensitive monomer 2-methyl-2-acrylate-2-(2-methoxyethoxy-ethyl) ethyl ester (MEO_2_MA) and oligoethylene glycol methyl ether methacrylate (OEGMA) as well as the pH-sensitive monomer *N*,*N*-Diethylaminoethyl methacrylate (DEAEMA) were used to make the gel with temperature and pH response. Four kinds of physicochemical double-crosslinked amphiphilic co-network gels with different polymerization degrees were prepared by the one-pot method using the stereocomplex between polylactic acid as physical crosslinking and click chemistry as chemical crosslinking. By testing morphology, swelling, thermal stability and mechanical properties, the properties of the four hydrogels were compared. Finally, the drug release rate of the four gels was tested by UV–Vis spectrophotometer. It was found that the synthetic hydrogels had a good drug release rate and targeting, and had great application prospect in drug delivery.

## 1. Introduction

According to surveys and studies, malignant tumors rank second among all diseases that cause human mortality [1]. Therefore, the treatment of malignant tumors has attracted great attention. However, one of the disadvantages of malignant tumor treatment is that large doses of chemotherapy can cause serious side effects [2]. Therefore, local treatment is a very important way to treat malignant tumors. Stimulus-responsive hydrogels are gradually coming into people’s view because of their three-dimensional network structure and their ability to change the pore size and thus the drug release rate in response to changes in the external environment. Stimulus-responsive hydrogels include temperature-responsive hydrogels [3,4], pH-responsive hydrogels [5,6], redox-responsive hydrogels [7,8] and photo-responsive hydrogels [9,10]. Temperature- and pH-responsive hydrogels are widely used in drug delivery systems because they can respond to the important physiological parameters of human body temperature and pH.

Hydrogels form network structures mainly through crosslinking, which mainly includes physical crosslinking and chemical crosslinking [11,12,13]. Among them, the formation of physical crosslinking bonds includes intermolecular hydrogen bond interactions [14], electrostatic coupling [15] and coordination bonds [16]. Chemical crosslinking is generally crosslinked by chemical covalent bonding, and the crosslinking methods include enzyme-induced cross-linking [17], free radical polymerization-induced crosslinking [18] and Schiff base generation [19].

Polylactic acid (PLA) is a hydrophobic polymer that can be used in biomedicine because it is a lactic acid derivative made from renewable substances, so it has good biocompatibility and degradability [20,21]. PLA has a left-handed (PLLA) and right-handed (PDLA) structure, which are pairs of enantiomers [22,23]. PDLA and PLLA can form physical crosslinking points through intermolecular hydrogen bonding [24]. However, conventional physically crosslinked gels suffer from poor stability and poor mechanical properties [25]. In order to improve the performance of the gel, chemical crosslinking is introduced into the gel. Click chemistry has the characteristics of high stability, high selective reactivity and biocompatibility, which is very suitable for application in the biomedical field. Among them, the Cu(I) catalytic click reaction of azide–acetylene cycloaddition has been widely used in the preparation of hydrogel networks due to its high yield, no side reactions and good functional group tolerance [25,26,27,28]. The temperature-sensitive monomer 2-methyl-2-acrylate-2-(2-methoxyethoxy) ethyl ester (MEO_2_MA) and oligoethylene glycol methyl ether methacrylate (OEGMA) are hydrophilic and biocompatible [29]. In addition, LCST of the polymer can be adjusted by changing the ratio between the two, so that the LCST value is close to the human body temperature [29,30,31]. *N*,*N*-Diethylaminoethyl methacrylate (DEAEMA) has pH sensitivity because of its tertiary amino structure, which can be protonated and deprotonated with the change in external environment [32,33]. These monomers have been used in the field of medicine because of their good biocompatibility [34,35].

In our previous work [36], the synthesized gels were fragile and had a low drug release rate of 18%, whereas the gels prepared in this study corrected the fragility of the gels and replaced the EDGMA with click chemistry bonds. The drug release rate of the gel was improved by replacing EDGMA with click chemistry [37,38], which is highly efficient, specific and bioorthogonal, which are all required for drug delivery [39]. Moreover, click chemistry has good degradability compared to EGDMA [26,40].

In the present study, a double-crosslinked amphiphilic co-network hydrogel with temperature and pH response was prepared with the aim of modulating the drug release rate of the gel by adjusting its structure. First, a series of macromolecular monomers HEME-PLLA_n_ and HEMA-PDLA_n_ with different polymerization degrees was produced by the ring-opening polymerization of 2-Hydroxyethyl methacrylate (HEMA) and lactide; then they were treated with azide and alkynylation, respectively. The experiment involved the addition of cuprous bromide, an azide and acetylene base reaction to form triazole chemical crosslinking and then an ultrasonic interaction occurred between the molecular hydrogen bonds to form physical crosslinking. Finally, the temperature-sensitive monomers MEO_2_MA and OEGMA and the pH-responsive monomer DEAEMA were used to prepare the gel by one-pot method. The chemical and physical crosslinking bonds of the hydrogels were characterized by near-infrared to mid-infrared spectroscopy (FT-IR) and powder X-ray diffractometer (XRD). The mechanical properties and drug-loading properties of hydrogels were tested by dynamic viscoelastic spectroscopy (DMA) and UV–Vis. It was found that the prepared amphiphilic co-network gels with temperature- and pH-responsive physical and chemical double crosslinking had a good prospect of sustained release. It was found that the prepared amphiphilic co-network gels with temperature- and pH-responsive physicochemical double cross-linking greatly increased the drug release rate compared to our previously investigated gels and already reported drug-loaded gels, which are promising for the sustained release of loaded substances.

## 2. Results and Discussion

### 2.1. Synthesis

**Scheme 1:**
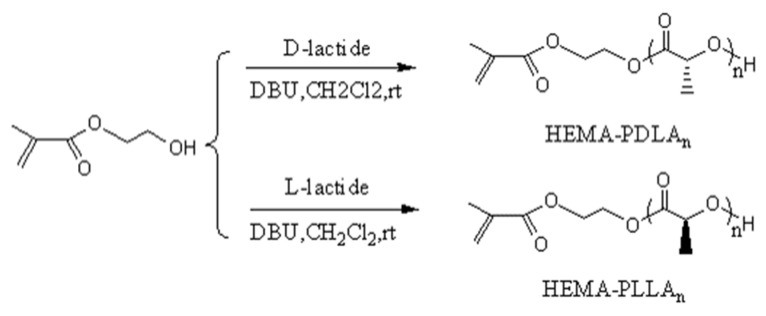
Synthesis of macromolecular monomer HEMA-PLLA_n_ and HEMA-PDLA_n_.

**Scheme 2:**
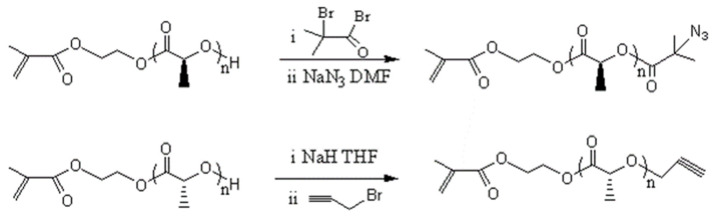
Synthesis of HEMA-PLLA-N_3_ and HEMA-PDLA-alkyne.

**Scheme 3:**
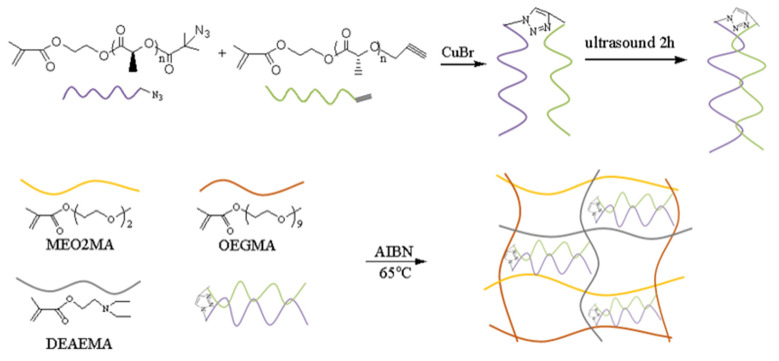
Synthesis of stereocomplex and click-chemical double-crosslinked gels.

The ring-opening polymerization of 2-Hydroxyethyl methacrylate (HEMA) and lactide was catalyzed by DBU to form HEME-PLLA_n_ and HEMA-PDLA_n_, The synthesis steps are shown in Scheme 1. Then 2-Bromoisobutyryl bromide and NaN_3_ were added to HEME-PLLA_n_ to form HEME-PLLA-N_3_. NaH and alkyne bromide were added to HEMA-PDLA_n_ to form HEMA-PDLA-alkyne. The synthesis steps are shown in Scheme 2. CuBr was added to react to the azide and alkyne to form triazole to form chemical cross-links, and ultrasonication was performed to create intermolecular hydrogen bonding to form physical crosslinks. Finally, the temperature-sensitive monomers MEO_2_MA and OEGMA, and the pH-responsive monomer DEAEMA were added to prepare the hydrogel by free radical polymerization by the one-pot method. The synthesis steps are shown in Scheme 3.

By adjusting the different dosage ratios of HEMA and lactide in macromolecular monomers, the degree of polymerization is controlled, and the influence of macromolecular monomers with different polymerization degrees on gel properties is studied. The specific dosage of the gels is shown in Table 1.

### 2.2. Structural Characterization

#### 2.2.1. Structural Characterization of HEMA-PLLA_n_ and HEMA-PDLA_n_

**Figure 1:**
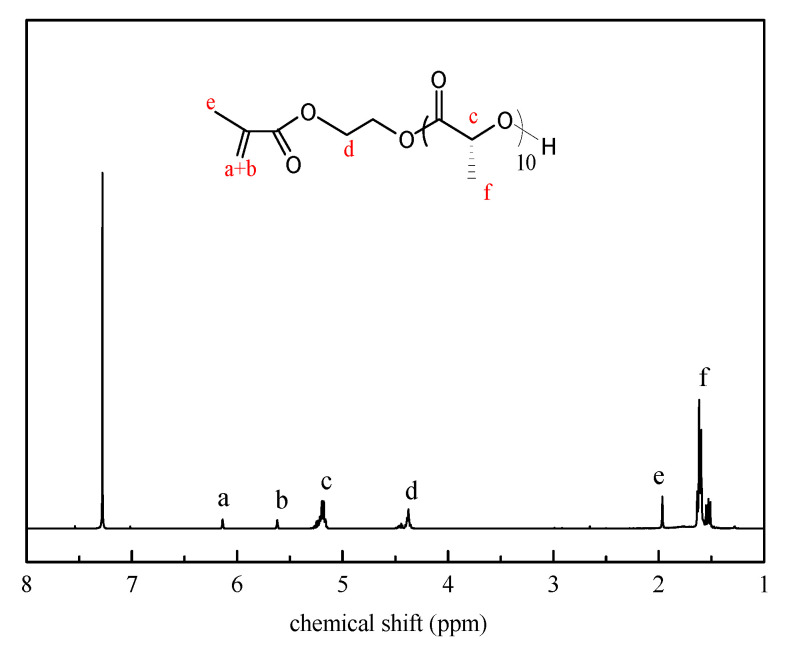
^1^H NMR diagram of HEMA−PLLA_10_.

Figure 1 shows the ^1^H NMR diagram of the macromolecular monomer HEMA-PLLA_10_. In the figure, 6.12 ppm and 5.60 ppm are the proton peaks of the carbon–carbon double bond of the macromolecular monomer, and 5.25–5.15 ppm and 1.54–1.62 ppm are the proton peaks of the submethyl and methyl groups in the LA repeating unit, respectively; 4.26–4.40 ppm is the proton peak of O=COCH_2_CH_2_O- group methylene, 1.95 ppm is the proton peak of -(CH_3_)C=CH_2_ group methyl. The figure illustrates the successful synthesis of macromolecular monomers. ^1^H NMR diagram of HEME-PLLA and HEMA-PDLA are the same, so we will not go into details here.

**Figure 2:**
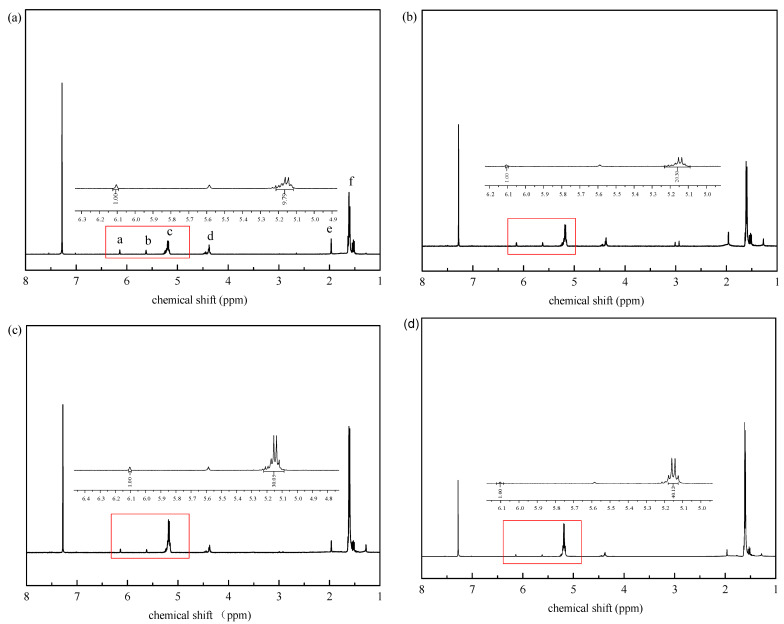
(**a**) ^1^H NMR diagram of HEMA−PLLA_10_; (**b**) ^1^H NMR diagram of HEMA−PLLA_20_; (**c**) ^1^H NMR diagram of HEMA−PLLA_30_; (**d**) ^1^H NMR diagram of HEMA−PLLA_40_.

The number of LA units *n* in HEMA-PLLA_n_ and HEMA-PDLA_n_ is calculated by the following formula:

(1) $$n=\frac{A_{c}}{A_{a}}$$

where *A_c_* and *A_a_* represent the integral area of c and a, respectively. Figure 2a, HEMA-PLLA_10_, *A_a_* = 1.00 and *A_c_* = 9.79, so *n* = 9.79 ≈ 10. Figure 2b, HEMA-PLLA_20_, *A_a_* = 1.00 and *A_c_* = 20.30, so *n* = 20.30 ≈ 20. Figure 2c, HEMA-PLLA_30_, *A_a_* = 1.00 and *A_c_* = 30.05, so *n* = 30.05 ≈ 30. Figure 2d, HEMA-PLLA_40_, *A_a_* = 1.00 and *A_c_* = 40.12, so *n* = 40.12 ≈ 40.

**Figure 3:**
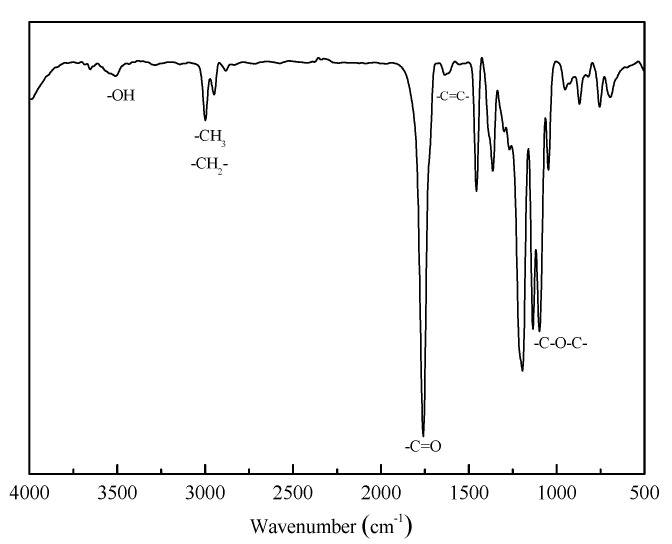
FT−IR spectrum of the macromolecular monomer.

Figure 3 shows the FT-IR spectrum of the macromolecular monomer. In the figure, the peaks at 1750 cm^−1^ and 3548 cm^−1^ are the stretching vibrations of C=O and -OH, respectively, and the peak at 1189 cm^−1^ is the C-O-C stretching vibration. The peak at 1631 cm^−1^ is the stretching vibration of -C=C-, and the peak at 3000 cm^−1^ is the bending vibration and stretching vibration of C-H of methyl and methylene.

#### 2.2.2. Structural Characterization of HEMA-PLLA-N_3_ and HEMA-PDLA-Alkyne

**Figure 4:**
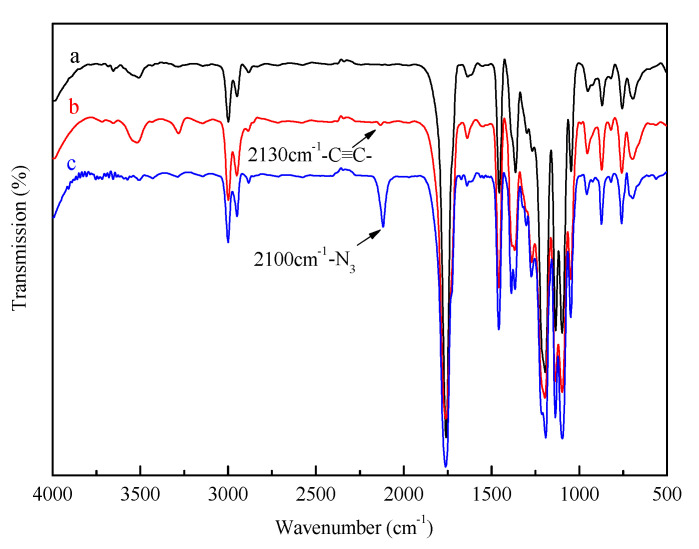
(a) FT−IR spectrum of the macromolecular monomer; (b) FT−IR spectrum of HEMA−PDLA−alkyne; (c) FT−IR spectrum ofHEMA−PLLA−N_3_.

Figure 4 shows the FT-IR spectra of the macromolecular monomer, HEMA-PDLA-alkyne, and HEMA-PLLA-N_3_, respectively. By comparing (a) with (b), it is found that a stretching vibration absorption peak of alkyne-C≡C- appears at 2130 cm^−1^, which indicates the successful synthesis of HEMA-PDLA-alkyne. By comparing (a) with (b), a stretching vibration absorption peak of azido-N_3_ was found at 2100 cm^−1^, indicating the successful synthesis of HEMA-PLLA-N_3_.

#### 2.2.3. Structural Characterization of Gels

**Figure 5:**
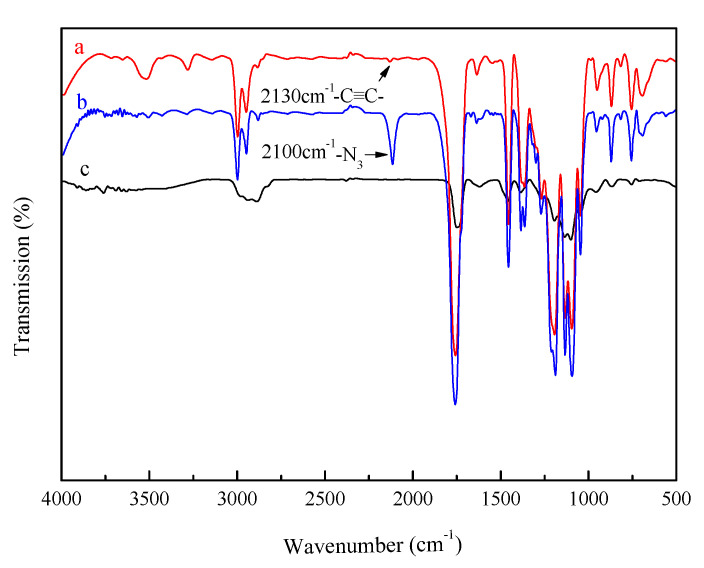
(a) FT−IR spectrum of HEMA−PDLA−alkyne; (b) FT−IR spectrum of HEMA−PLLA−N_3_; (c) FT−IR spectrum of gel.

Figure 5 shows the FT-IR spectra of HEMA-PDLA-alkyne, HEMA-PLLA-N_3_ and gel, respectively. Comparison of (c) with (a) and (b) reveals that both the azide and alkyne peaks disappear, indicating that the azide group reacted with the alkyne group and successfully clicked the chemical bond.

**Figure 6:**
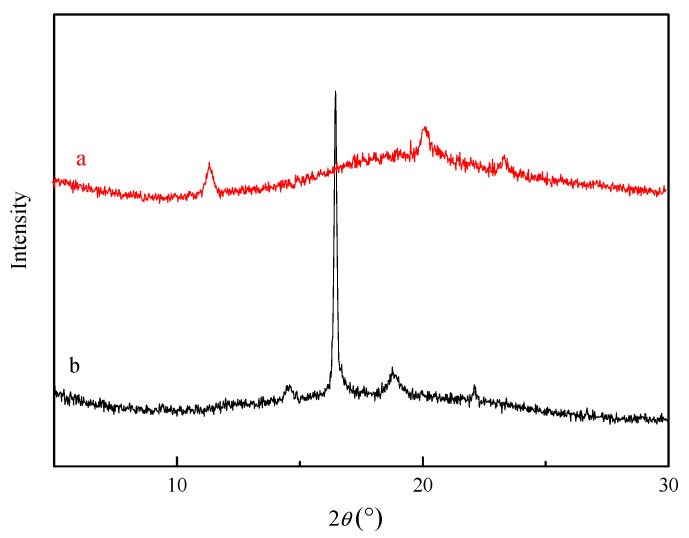
(a) XRD pattern of the gel; (b) XRD pattern of the macromolecular monomer.

Through sonication, the -CH_3_ of PLLA in HEMA-PLLA-N_3_ formed CH_3_···O=C hydrogen bonds with the C=O bonds of PDLA in HEMA-PDLA-alkyne, and the -CH_3_ of PDLA in HEMA-PDLA-alkyne formed CH_3_···O=C hydrogen bonds with the C=O bonds of PLLA in HEMA-PLLA-N_3_, which led to the formation of steric coordination compounds and the formation of physical crosslinks. Figure 6 shows the XRD patterns of gel and HEMA-PDLA. In (b), it can be seen that diffraction peaks appear when 2*θ* is 15.8°, 18.2° and 19.8°, which is the characteristic peak of macromolecular monomer, which can also prove that the successful synthesis of macromolecular monomer (a) is the XRD pattern of the gel. In the figure, the characteristic peaks of macromolecular monomers disappear, and the diffraction peaks with 2*θ* of 12°, 21° and 24° appear. The diffraction peaks are the characteristic peaks of the stereoscopic complexation of PLLA and PDLA, which can prove that the stereoscopic complexation of PLLA and PDLA is successful, and the gel forms a physical crosslinking bond. The XRD patterns of HEMA-PLLA and HEMA-PDLA are the same, and the XRD patterns of gels with different polymerization degrees are also the same, so we will not go into details here [41,42].

### 2.3. Morphology Analysis of Gels

**Figure 7:**
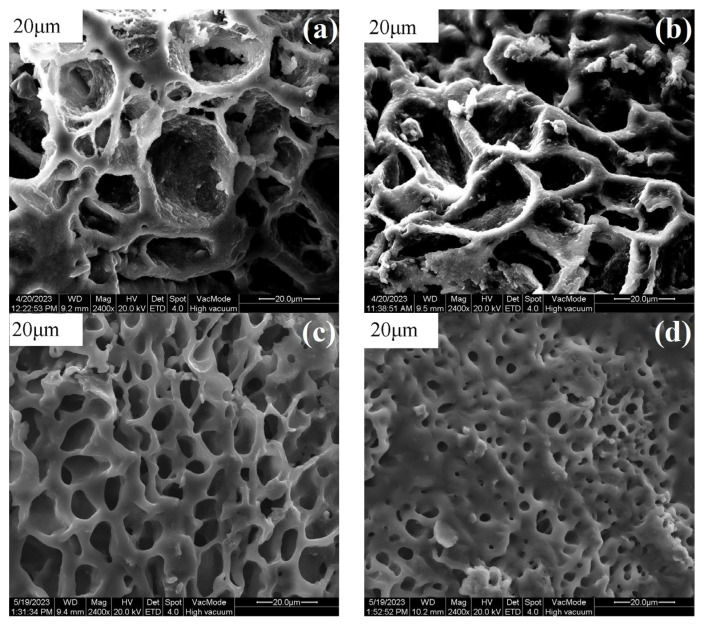
SEM of gel 1 (**a**); gel 2 (**b**); gel 3 (**c**); gel 4 (**d**).

Figure 7 shows SEM images of gels with different degrees of polymerization at 2400 magnification, from which it can be seen that all four gels have pore size structures, which provides the premise for gel drug loading. In addition, with the increase in the degree of polymerization, the pore size of the gel gradually becomes smaller, because the higher the degree of polymerization, the more crosslinking points that will be formed, the closer the internal structure of the gel and the pore size will be smaller [43,44].

### 2.4. The Amphiphilic Nature of Gels

**Figure 8:**
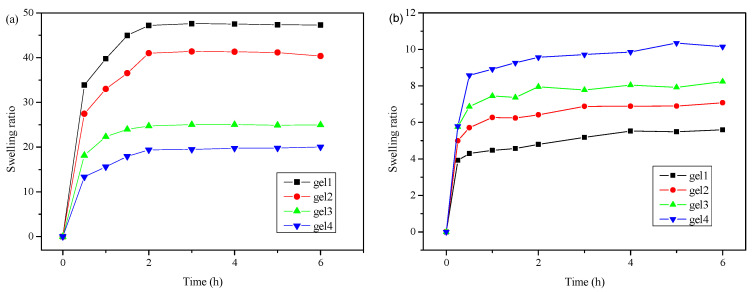
(**a**) Swelling rate of gels in distilled water at 25 °C; (**b**) swelling rate of gels at THF.

The gels with different degrees of polymerization were placed in distilled water and tetrahydrofuran solution at 25 °C. It can be seen from Figure 8 that gels can swell in both aqueous and organic phases, and the swelling trend increases first and then flattens out with the increase in time. In the water, the swelling rate of gel 1 is the largest, and the swelling rate of gel 2, gel 3 and gel 4 decreases successively. On the contrary, in the organic phase, the swelling rate of gel 4 is the largest and the swelling rate of gel 1 is the smallest. This is because, in the organic phase, swelling is partly related to the hydrophobic part of the gel, that is, it is related to the degree of polymerization of LA in the macromolecular monomer [45], the greater the degree of polymerization, the more repeat units, the more organic application points and the greater the swelling will be.

**Figure 9:**
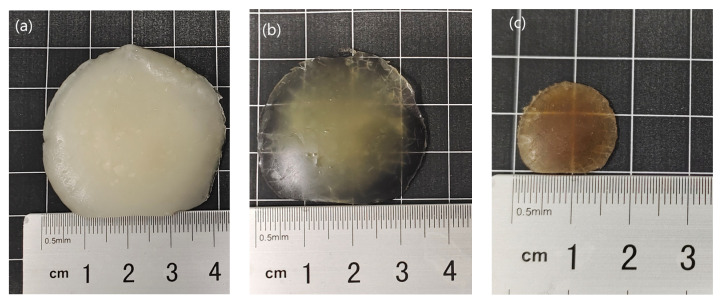
(**a**) Digital photo of the gel 1 completely swelling in distilled water; (**b**) digital photo of the gel 1 completely swelling in THF; (**c**) digital photo of dry gel 1.

Dried gel 1 was immersed in distilled water at 25 °C and after it was completely dissolved and equilibrated, it was photographed and recorded with a digital camera, as shown in Figure 9a. Then, gel 1 was dried in an oven at 70 °C, and the photos were recorded as shown in Figure 9c. Finally, the dried gel was soaked in the organic phase tetrahydrofuran, and the photos of its complete swelling are shown in Figure 9b. It can be seen from the figure that the gel can swell in both the organic phase and the aqueous phase, and the volume of swelling in the organic phase is smaller than that in the aqueous phase, which is consistent with the swelling rate diagram of the gel, and further proves that the synthetic physicochemical double-crosslinked gel has amphiphilic.

### 2.5. Temperature Sensitivity of the Gels and Reversibility

Buffer solutions can have some effect on LCST, and the interaction between water and buffer anions can cause a shift in the hydration layer. Additionally, the glycol group of POEGMA will be strongly bound to the salt cation (Na+) in the buffer solution. The addition of salt will shift the phase transition temperature linearly towards lower temperatures [46,47]. To attenuate this effect, the temperature sensitivity of the gels was tested using secondary distilled water at pH = 7 as a solvent.

**Figure 10:**
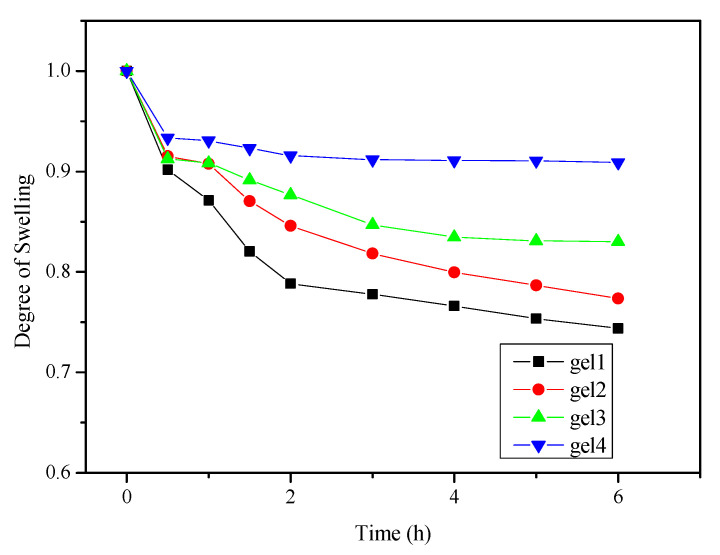
Deswelling rate of gels in distilled water at 37 °C.

The gel with different polymerization degree was put in 25 °C and 37 °C distilled water to test its swelling rate and deswelling rate. At 25 °C, when the temperature is lower than LCST of the temperature-sensitive monomer, the temperature-sensitive monomer forms a hydrogen bond with water, the gel aperture expands, water absorbs and is in a swelling state. The smaller the crosslinking density, the more porous the gel aperture, the more that water absorbs and the greater the swelling rate, as shown in Figure 8a. At 37 °C, the temperature is higher than the LCST of the temperature-sensitive monomer, and the hydrogen bond formed by thermosensitive monomer and the water molecule breaks the chain, resulting in gel water loss and deswelling [48]. The gel with smaller crosslinking density is more likely to collapse after external environment changes, and the deswelling rate is smallest, as shown in Figure 10.

The combination of oligoethylene glycol methacrylate with acrylamide tends to result in polymers with schizophrenic features, where most of the amide groups form hydrogen bonds with the ether groups of OEGMA, creating a more hydrophobic structure and affecting the LCST [49,50], but this phenomenon is controlled by the molar mass. In this study, the molar mass of both DEAEMA and OEGMA is very low, so we think the copolymer of MEO_2_ME and OEGMA has more influence on the regulation of LCST.

**Figure 11:**
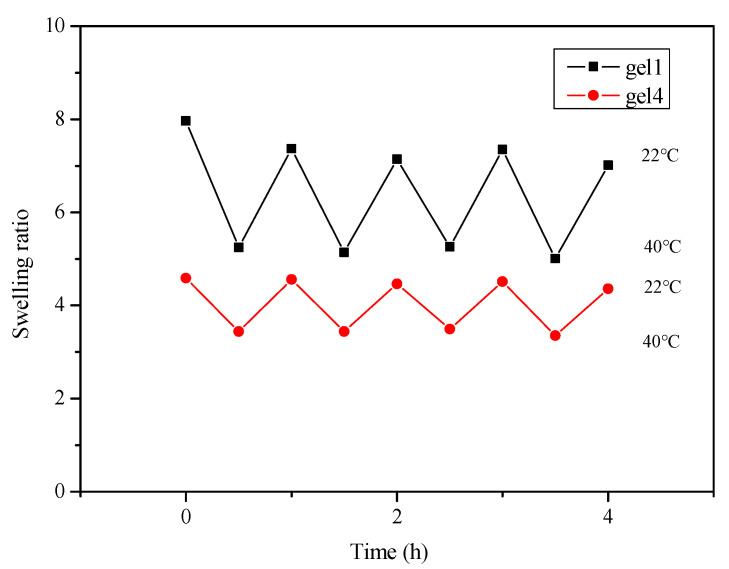
Reversible swelling of gel 1 and gel 4.

Gel 1 and gel 4 were placed in a water bath at 22 °C and 40 °C for half an hour to test their swelling rates, and Figure 11 was obtained. It can be seen from the figure that gel 1 and gel 4 have fixed swelling rates in the four cycles, indicating that the synthesized gel has good reversibility. When the external conditions change, it will not destroy the performance of the gel and has stability.

### 2.6. pH Sensitivity of the Gels

**Figure 12:**
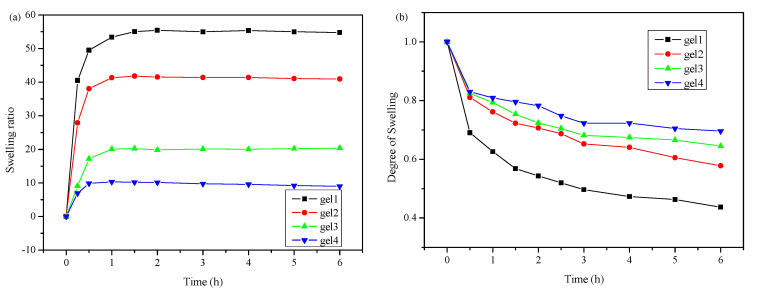
(**a**) Swelling rate of gels in solution at 37 °C and pH = 5; (**b**) deswelling rate of gels in solution at 37 °C and pH = 9.

**Figure 13:**
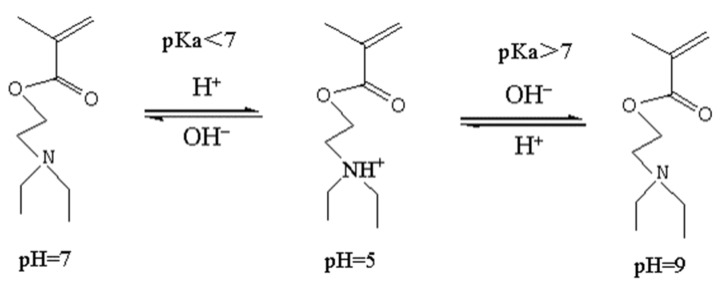
Molecular structural formulae of DEAEMA at different pH values.

The pH sensitivity of the gel was mainly related to DEAEMA. As shown in Figure 12a, when the temperature is 37 °C and pH = 5, the gel is in a swelling state. Because the p*K*_a_ value of DEAEMA is equal to 7, when the pH is less than 7, the tertiary amino group in DEAEMA is protonated, as shown in Figure 13, resulting in an electrostatic effect inside the gel, the gel pore size is enlarged, the gel absorbs water and the gel begins to swell. In addition, the larger the crosslinking density, the smaller the gel pore size, the less water absorption there is, and the smaller the swelling rate, so the swelling rate of gel 4 is the smallest. When pH = 9 is greater than p*K*_a_ value of DEAEMA, the tertiary amino group in DEAEMA is deprotonated, the internal electrostatic effect of the gel disappears [51,52], the gel loses water and is in a state of deswelling. In addition, the gel with higher crosslinking density is less likely to collapse after being subjected to changes in the external environment, and the deswelling rate is larger. Therefore, the deswelling rate of gel 4 is the largest, as shown in Figure 12b. The gels have good pH sensitivity, allowing the gel to have different drug release rates in different pH environments.

### 2.7. Thermal Properties Analysis of Gels

**Figure 14:**
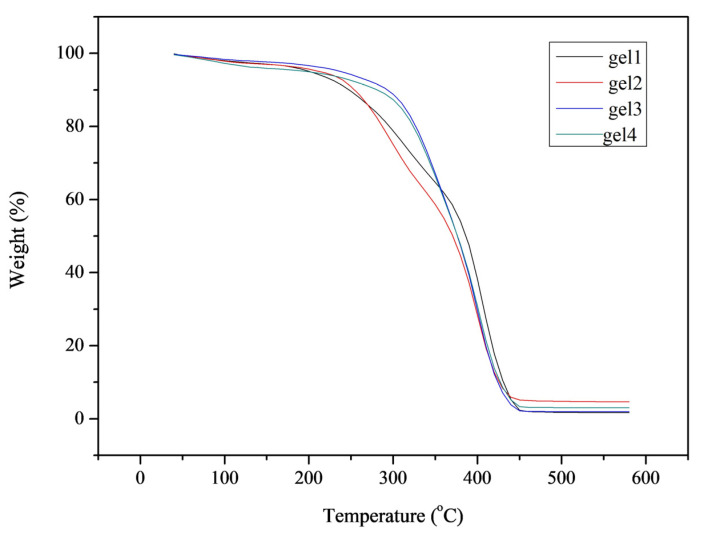
Thermal analysis diagram of gels.

**Figure 15:**
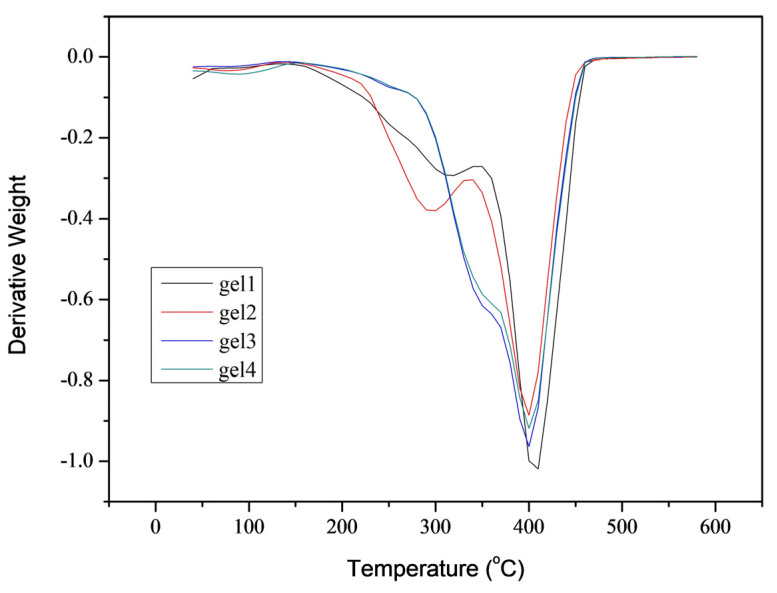
DTG diagram of gels.

The temperature at which the gel loses weight for the first time can reflect the thermal stability of the gel. From Figure 14 and Figure 15 and Table 2, it can be seen that gel 1 and gel 2 showed two significant weight losses, while gel 3 and gel 4 showed almost one weight loss. This shows that the thermal stability of gel 3 and gel 4 is higher than that of gel 1 and gel 2 [43,53]. This is because the higher the degree of polymerization, the more hydrogen bonding, the stronger the force and the higher the relative weight loss temperature [45]. In Figure 15, there is a drop at around 100 °C, which we believe is caused by the small amount of free water in the sample. In addition, gel 2 had a minimal mass loss at the end of 600 °C, which we believe is due to the small amount of copper ions used for click chemistry remaining in the sample, which may have an effect on weight loss.

### 2.8. Analysis of Mechanical Properties of Gels

**Figure 16:**
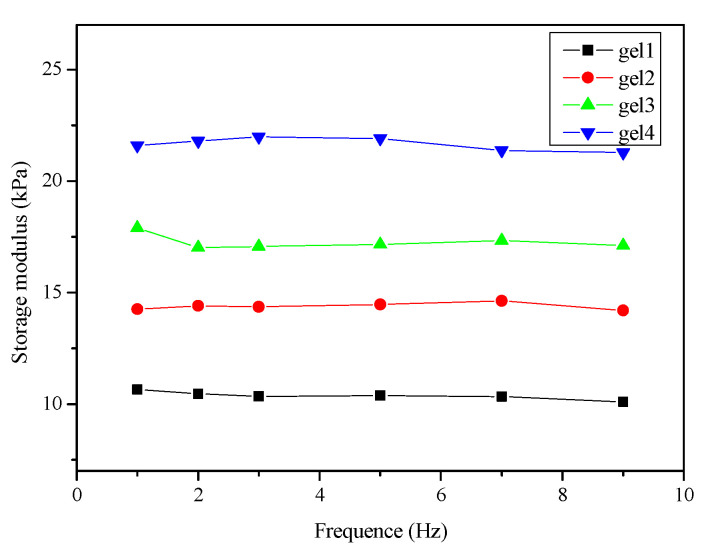
Energy storage modulus curve of gels.

The loss tangent and energy storage modulus of four kinds of gels were measured by dynamic viscoelastic spectrometer. The energy storage modulus can reflect the elasticity of the material, and the greater the energy storage modulus, the greater the elasticity. As shown in Figure 16, with the increase in polymerization degree, the gel hydrogen bond force is stronger, the internal structure of the gel is more compact and the energy storage modulus is larger [44], and the mechanical properties are better. The tangent value of the loss angle reflects the viscoelasticity of the material. The smaller the tangent value of the loss angle, the greater the elasticity, and the larger the tangent value of the loss angle, the greater the viscosity. The loss angle tangent values of gel 1, gel 2, gel 3 and gel 4 at 9 Hz are 0.111, 0.0607, 0.0562 and 0.0565, respectively. The loss angle tangent values of the four gels are all small, indicating that the synthesized gels are mainly elastic under external forces. Compared to most physically crosslinked hydrogels, the mechanical properties of the gels in this study were improved [54,55]. Because we added chemical crosslinking to improve the mechanical properties of the gel, compared to a portion of physicochemical double-crosslinked hydrogels, the gels in this study have a lower energy storage modulus [26,56]; we think this is because the addition of the pH-sensitive monomer increases the swelling of the gel, which reduces the elasticity of the gel.

### 2.9. Sustained Drug Release of the Gels

**Figure 17:**
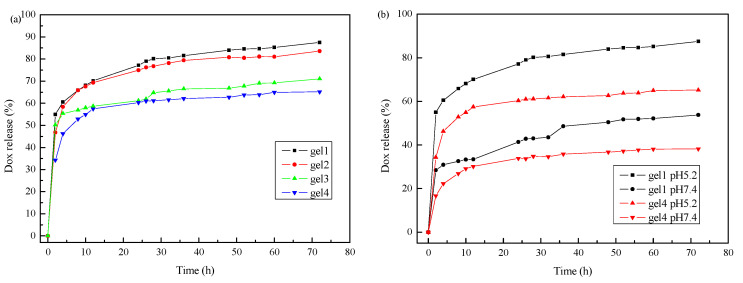
(**a**) Drug release rate of the gel at pH = 5.2, 37 °C; (**b**) drug release rates of gel 1 and gel 4 at pH = 7.4, 37 °C and pH = 5.2, 37 °C.

The environment around the tumor tissue is weakly alkaline and pH is about 5.2, so four kinds of gels were put into an environment of 37 °C and pH = 5.2 to simulate the physiological parameters of the tumor tissue. Drug release studies were conducted on the four gels, as shown in Figure 17a. It was found that the release rate of gel 1, gel 2, gel 3 and gel 4 after 72 h was 87.4%, 83.5%, 80.0% and 65.1%. Thus, the four gels all have good drug release rates, and the larger the degree of polymerization, the smaller the gel aperture and the less drug release. The prepared double-crosslinked gels greatly improved the drug release rate compared to our previously studied gels [36] and drug-carrying gels that have been reported [57,58].

In addition, gel 1 and gel 4 were placed in an environment of 37 °C and pH = 7.4 to simulate human physiological parameters and explore the effect of pH on gel drug release, as shown in Figure 17b. The drug release rate of gel 1 and gel 4 in a pH = 7.4 environment was lower than that in a pH = 5.2 environment. These results indicated that the synthetic double-crosslinked gel had good pH response, and the drug release rate in the pH 7.4 environment was lower than that in the pH 5.2 environment, indicating that the drug could act on tumor tissues faster and had certain targeting properties, which was of great significance in gel drug delivery.

## 3. Conclusions

In this paper, the physical and chemical double-crosslinked hydrogels were formed by the stereoscopic complexation and click chemistry between polylactic acid. The thermosensitive monomers MEO_2_MA and OEGMA and the pH-sensitive monomer DEAEMA with good biocompatibility were added to make the gel have temperature and pH responsiveness. Through environmental scanning electron microscopy, it was found that the synthesized hydrogels had uniform pore size, which provided the possibility for gel drug loading. Through sustained drug release studies, it was found that the synthetic hydrogel had a good drug release rate and targeting. The results indicate that the prepared physicochemical double-crosslinked gels with temperature and pH response have great application prospects in drug delivery.

## 4. Materials and Methods

### 4.1. Materials

Hydroxyethyl methacrylate (HEMA, 99%), 1, 8-diazabicyclic [5.4.0] undeca-7-ene (DBU, 99%) and 2-bromoisobutanoyl bromide (BIBB, 98%) were purchased from Beijing Balinway Technology Co., Ltd., (Beijing, China). L-lactide (L-LA, 98%), 2-methyl-2-acrylate-2 -(2-methoxy-ethoxy) ethyl ester (MEO_2_MA, 97%, *M*_n_ = 188.22 g·mol^−1^), oligoethylene glycol methyl ether methacrylate (OEGMA, 95%, *M*_n_ = 475 g·mol^−1^) and azodiisobutyronitrile (AIBN, 98%) were purchased from Shanghai Maclin Biochemical Technology Co., Ltd., (Shanghai, China). D-lactide (D-LA, 99%) was purchased from Aladdin Chemicals Co., Ltd., (Shanghai, China). Triethylamine (TEA, 99%), sodium azide (analytical pure) and sodium hydride (analytical pure) were purchased from Shanghai Sinophosphoric Chemical Reagent Co., LTD., (Shanghai, China). *N*,*N*′-dimethylformamide (DMF) ultra-dry solvent and tetrahydrofuran (THF) ultra-dry solvent were purchased from Anaeji Chemical Co., Ltd., (Shanghai, China). Dichloromethane (DCM) was purified, and the experimental water was secondary distilled water.

### 4.2. Synthesis

#### 4.2.1. Synthesis of Macromolecular Monomers HEMA-PLLA_n_ and HEMA-PDLA_n_

Using the synthesis of macromolecular monomers HEMA-PLLA_30_ and HEMA-PDLA_30_ as an example, L-lactone (0.5 g, 3.47 mmol) and D-lactone (0.5 g, 3.47 mmol) were added into the flask, and then initiator HEMA (28 μL, 0.23 mmol) and 30 mL dichloromethanes were added into the flask, followed by a dry nitrogen and stirring reaction. After 5 min, 50 μL DBU catalyst was added. After the reaction at room temperature for 12 h, benzoic acid was added to terminate the reaction. The resulting solution was precipitated in 30 mL of n-hexane in an ice bath. Finally, the macromolecular monomers HEMA-PLLA_30_ and HEMA-PDLA_30_ were obtained by drying them in a fume hood.

#### 4.2.2. Synthesis of HEMA-PLLA-N_3_

This process involved adding 0.5 g of HEMA-PLLA30 macromolecular monomer to the flask and dissolving it in 5 mL of DMF, then passing it through the nitrogen and adding 120 mL of the acid-binding agent triethylamine. After half an hour, 250 mL of 2-bromoisobutyryl bromide was added in an ice bath and the reaction was stirred for 24 h. Then, 0.20 g of sodium azide was dissolved in a small amount of distilled water and drops were added to the flask for 48 h. Finally, the obtained solution was put into a dialysis bag with a molecular weight of 500 for impurity removal and purification for 48 h, and the water in the solution was removed by a freeze dryer to obtain white powder HEMA-PLLA-N_3_.

#### 4.2.3. Synthesis of HEMA-PDLA-Alkyne

The macromolecular monomer containing 0.5 g of HEMA-PDLA_30_ was added to the flask, dissolved with 5 mL THF, had 35 mg of sodium hydride added, and half an hour later, had 300 μL of propyl bromide added for 48 h. Finally, the obtained solution was loaded into a dialysis bag with a molecular weight of 500 g and was purified for 48 h. The water in the solution was removed by a freeze dryer to obtain yellow colloidal HEMA-PDLA-alkyne.

#### 4.2.4. Synthesis of Physicochemical Double-Crosslinked Hydrogels

The synthetic HEMA-PLLA-N_3_, HEMA-PDLA-alkyne and DMF were added successively in a 10 mL round-bottomed flask, where the mass ratio of HEMA-PLLA-N_3_ and HEMA-PDLA-alkyne was 4:5, and the reaction system was sealed with rubber plugs. The pumping–nitrogen filling–pumping process was repeated three times in order to create an oxygen-free environment in the flask, followed by the rapid addition of CuBr for the click reaction. After drying the solvent in the flask with a rotary evaporator, 0.10 g of the product was weighed into a round-bottomed flask and 2 mL of THF was added and sonicated for two hours to make the PDLA and PLLA in the product stereocomplex, and then the solvent in the flask was dried again with a rotary evaporator. The temperature-sensitive monomer containing 235 μL of MEO_2_MA (5.74 mol/L), 65 μL of OEGMA (2.31 mol/L), 60 μL of pH-sensitive monomer DEAEMA (5 mol/L) and 0.0020 g of AIBN were added into the bottle, and deoxygenated three times with the pumping—nitrogen filling—pumping process, and then placed in the oil bath at 65 °C for 4 h to form a gel. The resulting gel was soaked in distilled water to remove impurities.

### 4.3. Methods

#### 4.3.1. Structural Characterization

The macromolecular monomers HEMA-PLLA_n_ and HEMA-PDLA_n_ were synthesized by using deuterated chloroform as solvent using a 400 MHz (JEOL) nuclear magnetic resonance spectrometer model JNM-ECZ400S/L1. After grinding and drying the sample with potassium bromide, the macromolecular monomers HEMA-PDLA-alkyne and HEMA-PLLA-N_3_, and the double-crosslinked gels were characterized by a Tensor II NIR spectrometer. Macromolecular monomers and double-crosslinked gels were tested using a powder X-ray diffractometer model Bruker D8 Advance at a voltage of 40 kv and a current of 40mA, with a scanning range of 5°–30° and a scanning speed of 2°/min.

#### 4.3.2. Morphological Testing of Gels

The gel, which fully swelled and balanced in the distilled water, was rapidly frozen with liquid nitrogen, and then dried with a freeze dryer. After spraying gold on the surface for 30 s, the morphology of the double-crosslinked gel was analyzed by an environmental scanning electron microscope (SEM) model Quanta 200.

#### 4.3.3. The Amphiphilic Nature of Gels

After freeze drying, the prepared series of gels were cut and weighed, and then put into distilled water at 25 °C and tetrahydrofuran solution. After removing them at a fixed time, the liquid on the gel surface was absorbed with filter paper, and weighed and recorded. Measurements were repeated 3 times and average results were reported. The swelling rate of the hydrogel can be calculated by the following formula:

*Swellingratio* = (*W_t_* − *W_d_*)/*W_d_* (2)

where *W_t_* is the mass (g) of the gel at the time of swelling to *t*, and *W_d_* is the mass (g) of the dried gel.

#### 4.3.4. Temperature Sensitivity of the Gels and Reversibility

The temperature sensitivity of the gel can be shown by the swelling rate and deswelling rate of the gel under different temperature conditions.

A certain amount of the dried gel was cut, weighed and recorded. The external temperature was adjusted to 37 °C, and then it was removed at regular intervals, filter paper was used to absorb the liquid on the gel surface, and it was weighed with an analytical balance. Measurements were repeated 3 times and average results were reported. The deswelling rate of the hydrogel can be calculated by the following formula:

*Deswellingratio* = (*W_t_* − *W_d_*)/*W_s_* (3)

where *W_t_* is the gel mass (g) at the time of swelling to *t*, *W_d_* is the gel mass (g) after drying, and *W_s_* is the gel mass (g) after complete swelling.

Gel 1 and gel 4 were swelled and balanced in distilled water at 22 °C and weighed, transferred to distilled water at 40 °C and weighed after half an hour, then transferred to solution at 22 °C and weighed after half an hour, and so on for four times to calculate the swelling rate and test the reversibility of the gels.

#### 4.3.5. pH Sensitivity of the Gels

The pH sensitivity of the gel can be shown by the swelling rate and deswelling rate of the gel under different pH conditions.

The processed involved weighing a certain quality of dried gel, putting it into a solution of 37 °C pH = 5, removing it and weighing it at regular intervals and calculating its swelling rate through Formula (2). The gel that was completely balanced in a pH = 5 solution was removed and placed into a pH = 9 solution, its mass was weighed at different times, and its deswelling rate was calculated by Formula (3).

#### 4.3.6. Thermal Properties Analysis of Gels

The Q-600 thermal analysis system was used to test the thermal stability of double-crosslinked gels with different polymerization degrees. This involved weighing about 5 mg of dried gel, placing it into an aluminum pot and scanning it in a nitrogen atmosphere at a heating rate of 10 °C/min and a temperature range of 40–600 °C.

#### 4.3.7. Analysis of Mechanical Properties of Gels

A dynamic viscoelastic spectrometer Q850 was used to measure the energy storage modulus and loss angle tangent of double-crosslinked gels with different polymerization degrees. Before the test, the gel was soaked in 37 °C of distilled water for more than 12 h to make it fully swollen. The test was performed with an oscillatory frequency sweep at a temperature of 37 °C, a force of 0.01 N and an amplitude of 20 μm.

#### 4.3.8. Sustained Drug Release of the Gels

Preparation of drug-loading gel: 5 mg of doxorubicin hydrochloride was weighed and added to 0.05 g of ultrasonic dried product, 118 μL of MEO_2_MA, 33 μL of OEGMA, 30 μL of DEAEMA, and the drug-loading gel was synthesized by the one-pot method.

Determination of the standard curve: doxorubicin hydrochloride solution with concentration of 50 μg·mL^−1^ was prepared, and PBS buffer was used as the blank control group. The UV–Vis spectrophotometer model UV-1901 was used for spectral scanning of 190–600 nm, and the maximum absorption wavelength was measured at 233 nm. A series of doxorubicin hydrochloride solution was prepared in the range of 1–50 μg·mL^−1^. The absorbance of doxorubicin hydrochloride was measured by photometry at 233 nm. The standard curve of doxorubicin hydrochloride was drawn by plotting. The fitted linear regression equation is as follows:

*Abs* = 0.0593*c* + 0.0434, *R*^2^ = 0.9989 (4)

where *Abs* is the absorbance of doxorubicin hydrochloride at a wavelength of 233 nm, and *c* is the concentration of doxorubicin hydrochloride.

In vitro release of drug-loaded gels: the prepared gels with different polymerization degrees were placed in beakers containing 200 mL of PBS buffer solution with pH = 7.4 and pH = 5.2. The process involved placing the beaker into a 37 °C gas bath thermostatic shaker, removing it at regular intervals, absorbing 3 mL of solution at a 233 nm wavelength to test absorbance and adding 3 mL of fresh PBS buffer with pH = 5.2 or pH = 7.4. The corresponding concentration can be calculated according to the standard curve of doxorubicin hydrochloride. Finally, the cumulative amount of drug in the gel can be calculated by the following formula:

(5) $$Cumulative\;release\left(\%\right)=\frac{V_{e}\sum_{1}^{n-1}C_{i}+{V_{0}C}_{n}}{m_{drug}}\times 100$$

where *V_e_* is the volume of solution obtained from the PBS buffer solution each time, that is, 3 mL, *V*_0_ is the total volume of the released solution, 200 mL; *C_i_* is the concentration of the gel released when the solution is removed for the first time; *m*_drug_ is the total mass of doxorubicin hydrochloride loaded into the gel, 5 mg.

## Figures and Tables

**Table 1:** Synthetic data of amphiphilic conetwork gels.

Samples	HEMA/PDLA (*n*:*n*)	HEMA/PLLA (*n*:*n*)	HEMA-PDLA-Alkyne/HEMA-PLLA-N_3_ (*w*:*w*)
gel 1	1:10	1:10	5:04
gel 2	1:20	1:20	5:04
gel 3	1:30	1:30	5:04
gel 4	1:40	1:40	5:04

The molar ratio of MEO_2_MA to OEGMA is 9:1; the molar ratio between DEAEMA and p(MEO_2_MA-*co*-OEGMA) is 1:5.

**Table 2:** Thermal degradation temperature and weight loss of gels.

Samples	Step	T_i_ (°C)	T_m_ (°C)	T_f_ (°C)	Mass Loss (%) at T_f_
gel 1	1	150	315	349	64.67
	2	355	412	450	2.34
gel 2	1	190	298	340	61.71
	2	350	403	445	5.176
gel 3	1	220	400	460	2.028
gel 4	1	222	403	460	3.148

## Data Availability

Not applicable.
